# Comparative effects of incretin-based therapy on doxorubicin-induced nephrotoxicity in rats: the role of SIRT1/Nrf2/NF-κB/TNF-α signaling pathways

**DOI:** 10.3389/fphar.2024.1353029

**Published:** 2024-02-19

**Authors:** Sandy R. Botros, Asmaa I. Matouk, Amr Amin, Gehan H. Heeba

**Affiliations:** ^1^ Department of Pharmacology and Toxicology, Faculty of Pharmacy, Minia University, El-Minia, Egypt; ^2^ College of Medicine, University of Sharjah, Sharjah, United Arab Emirates

**Keywords:** doxorubicin, nephrotoxicity, incretins, glucagon-like peptide-1, dipeptidyl peptidase-4 inhibitor

## Abstract

**Introduction:** Nephrotoxicity represents a major complication of using doxorubicin (DOX) in the management of several types of cancers. Increased oxidative stress and the activation of inflammatory mediators play outstanding roles in the development of DOX-induced kidney damage. This study aimed to investigate whether the two pathways of incretin-based therapy, glucagon-like peptide-1 receptor agonist (presented as semaglutide, SEM) and dipeptidyl peptidase-4 inhibitor (presented as alogliptin, ALO), differentially protect against DOX-induced nephrotoxicity in rats and to clarify the underlying molecular mechanisms.

**Methods:** Adult male rats were divided into six groups: control (received the vehicle), DOX (20 mg/kg, single I.P. on day 8), DOX + ALO (20 mg/kg/day, P.O. for 10 days), DOX + SEM (12 μg/kg/day, S.C. for 10 days), ALO-alone, and SEM-alone groups. At the end of the study, the animals were sacrificed and their kidney functions, oxidative stress, and inflammatory markers were assessed. Kidney sections were also subjected to histopathological examinations.

**Results:** The co-treatment with either ALO or SEM manifested an improvement in the kidney functions, as evidenced by lower serum concentrations of creatinine, urea, and cystatin C compared to the DOX group. Lower levels of MDA, higher levels of GSH, and increased SOD activity were observed in either ALO- or SEM-treated groups than those observed in the DOX group. DOX administration resulted in decreased renal expressions of sirtuin 1 (SIRT1) and Nrf2 with increased NF-κB and TNF-α expressions, and these effects were ameliorated by treatment with either ALO or SEM.

**Discussion:** Co-treatment with either ALO or SEM showed a renoprotective effect that was mediated by their antioxidant and anti-inflammatory effects via the SIRT1/Nrf2/NF-κB/TNF-α pathway. The fact that both pathways of the incretin-based therapy demonstrate an equally positive effect in alleviating DOX-induced renal damage is equally noteworthy.

## 1 Introduction

The number of cancer patients worldwide is rapidly increasing as it was reported that more than 19 million people have been diagnosed with cancer in 2020 (Ferlay et al., 2021; Global cancer Observatory, 2023). Doxorubicin (DOX) is an anthracycline anticancer agent which is widely used in clinical practice. It treats a variety of tumors, including breast cancer, lung cancer, prostate cancer, liver cancer, Kaposi’s sarcoma, osteosarcomas, and hematological malignancies, such as leukemia and lymphomas (Carvalho et al., 2009; Alghorabi et al., 2019). Nevertheless, DOX’s clinical application is linked to dose-dependent adverse effects on the kidney, heart, and blood, posing a significant clinical challenge that could curtail its effectiveness. It was reported that DOX can cause nephrotic syndrome and acute renal damage (with an incidence ratio of 4.5%) that may persist after drug discontinuation. It can damage the glomeruli and the tubules in addition to causing interstitial inflammation and fibrosis (Carvalho, et al., 2009; Carron et al., 2014; Yemm et al., 2019; Chang et al., 2023). The exact mechanism underlying DOX-induced nephropathy is not fully established. However, many studies reported that oxidative stress, mitochondrial dysfunction, and inflammation are involved in DOX-induced renal damage (Rashid et al., 2013).

Incretin-based therapies, such as glucagon-like peptide-1 receptor agonists (GLP-1RAs) and dipeptidyl peptidase-4 (DPP-4) inhibitors, have become crucial in the treatment of type 2 diabetes mellitus. However, recent studies have revealed the pleiotropic effects of incretin-based therapies beyond the glycemic control, which includes antioxidant, anti-inflammatory, and immunomodulatory effects (Radbakhsh et al., 2021; Yaribeygi et al., 2021). Furthermore, these treatments have displayed nephroprotective properties in diabetic patients (Vaghasiya et al., 2011; Muskiet et al., 2014; Esaki et al., 2017; Yaribeygi et al., 2021; Youssef et al., 2021). Additionally, their beneficial effects on kidney protection extend beyond diabetic models, as demonstrated by their ability to mitigate cisplatin-induced nephropathy (Katagiri et al., 2013; Iwakura et al., 2020) in various experimental contexts. This study aimed to explore the potential protective effects of semaglutide, a GLP-1RA, and alogliptin, a DPP-4 inhibitor, against DOX-induced nephrotoxicity. Additionally, it sought to elucidate the signaling pathways that might be responsible for these observed effects.

## 2 Materials and methods

### 2.1 Materials

DOX was obtained from Novartis, Switzerland. Alogliptin was obtained from Hikma Pharmaceuticals, London, United Kingdom. Semaglutide was obtained from Novo Nordisk, Denmark. Carboxymethyl cellulose sodium salt (CMC) was purchased from Oxford Lab Chem, India.

### 2.2 Animals

Adult male Wistar rats (160–220 g) were obtained from the Helwan Farm belonging to VACSERA Co., Cairo, Egypt. The animals were exposed to dark/light cycles of 12 h with free access to food and water and were kept for 2 weeks for acclimatization. All the experiments have been approved by the Research Ethics Committee for the laboratory animal’s care and use, Faculty of Pharmacy, Minia University, under the approval number: MPEC (230105).

### 2.3 Study design

**FIGURE 1:**
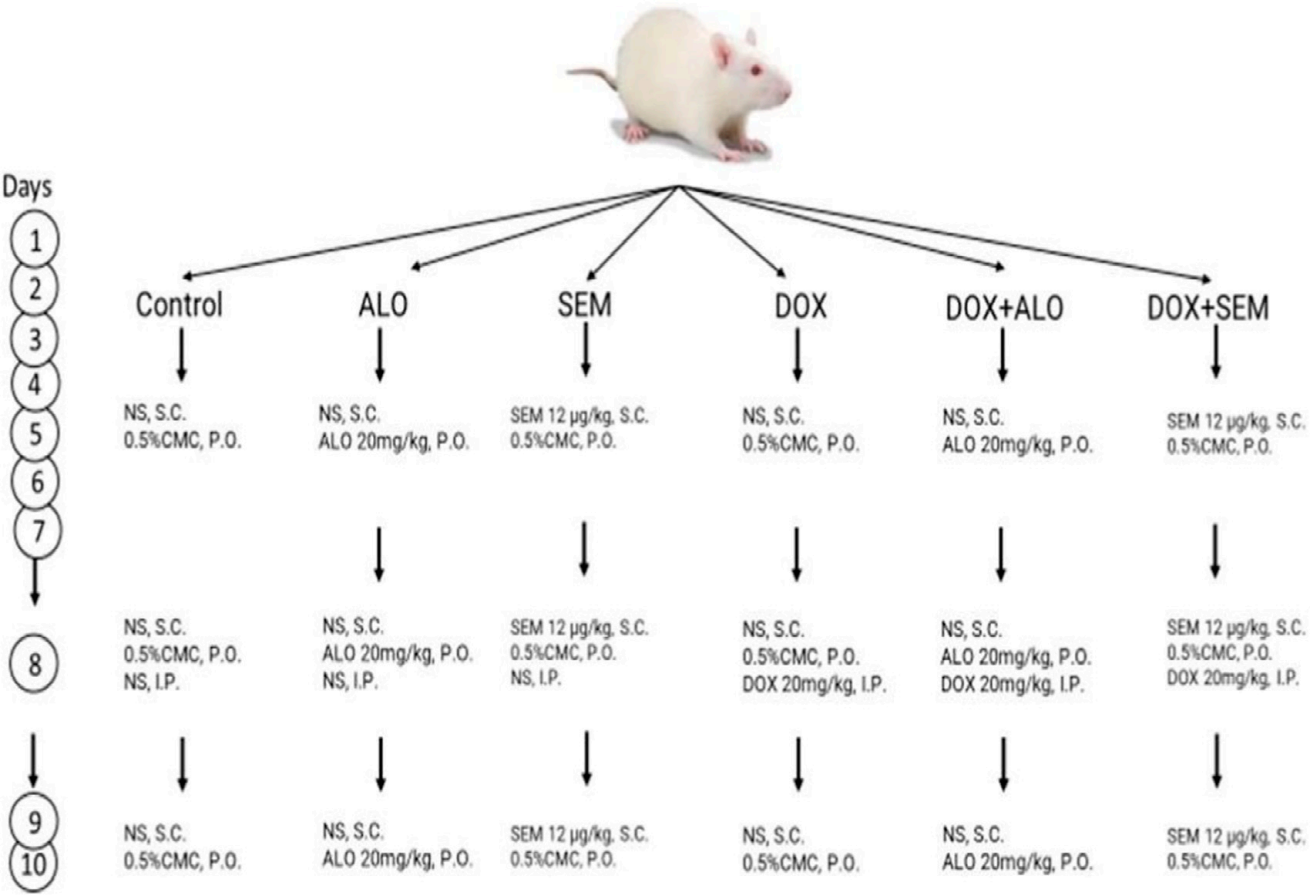
Experimental design.

The rats were randomly divided into six groups (*n* = 6, each), as summarized in Figure 1.

1-Control group: The rats were subcutaneously injected with normal saline and received 0.5% w/v carboxymethyl cellulose, the vehicle for alogliptin orally for 10 days. They were injected with saline I.P. on day 8.
2-Alogliptin (ALO) group: The rats received alogliptin (20 mg/kg/day, P.O.) suspended in 0.5% CMC for 10 days and normal saline I.P. on day 8.
3-Semaglutide (SEM) group: The rats received semaglutide (12 μg/kg/day, S.C.) for 10 days and normal saline I.P. on day 8.
4-Doxorubicin (DOX) group: The rats were given a single dose of DOX (20 mg/kg/day, I.P.) on day 8 and received the vehicles of semaglutide and alogliptin for 10 days.
5-Doxorubicin and alogliptin group (DOX + ALO): The rats received alogliptin (20 mg/kg/day, P.O.) for 10 days and DOX (20 mg/kg/day, I.P.) on day 8.
6-Doxorubicin and semaglutide group (DOX + SEM): The rats received semaglutide (12 μg/kg/day, S.C.) for 10 days and DOX (20 mg/kg/day, I.P.) on day 8.

The DOX, ALO, and SEM dosing regimen utilized in this study has been employed by other investigators (Kabel 2018; Qian et al., 2020; Al-Kuraishy et al., 2022) and also in the pilot experimental trials of the present study.

### 2.4 Blood and tissue collection

Twenty-four hours after the last dose, the rats were anesthetized using (1 g/kg) urethane by intraperitoneal injection after measuring the body weight, and then, blood samples were collected by decapitation and centrifuged at 3,500 rpm for 10 min for the separation of the serum. Fresh serum samples were used for the assessment of kidney function tests (creatinine and urea concentration). Other serum samples were stored at −80°C for the assessment of the cystatin C concentration. The kidneys were rapidly excised and washed in cold saline. The separated kidneys were dried on filter paper and weighed, and the kidney index was calculated according to the following equation: $Kidney\;index\;\left(g/100\;g\right)=\left(\frac{total\;kidneys\;weight}{body\;weight\;on\;sacrifice\;day}\right)\times 100$ (Eleazu et al., 2013).

Sections of the left kidney of each group were preserved in 10% formalin, embedded in paraffin blocks, and then sectioned into 4–6-μm-thick sections and stained with hematoxylin and eosin (H&E) for histopathological evaluations, according to Bancroft and Gamble (2008).

The rest of the kidneys were directly frozen in liquid nitrogen and kept at −80°C until the time of the analysis. Kidney tissues were homogenized in cold PBS and centrifuged at 10,000 rpm for 10 min, and the supernatant was used for biochemical analyses.

### 2.5 Assessment of renal functions

Serum levels of creatinine, urea, and cystatin C were used to evaluate the nephrotoxicity induced by DOX. Creatinine and urea concentrations were assessed spectrophotometrically using commercially available kits (Biodiagnostic, Cairo, Egypt), while the cystatin C concentration was measured using the commercially available ELISA Kit from Elabscience Biotechnology Co., Ltd., Wuhan, China; catalog no. E-EL-R0304.

### 2.6 Assessment of oxidative stress markers

The concentration of malondialdehyde, which is a product of lipid peroxidation, was used as a marker of oxidative stress. It was measured using the TBARS method described by Buege and Aust (1978). In addition, reduced glutathione and superoxide dismutase were also measured as antioxidant markers. Reduced glutathione was measured using the method described by Ellman (1959), while SOD activity was measured according to the method described by Marklund (1985).

### 2.7 Assessment of the renal expression of Nrf2, sirtuin 1, NF-κB, and TNF-alpha using Western blotting

Kidney tissues were homogenized in ice-cold Tris buffer and the protease inhibitor cocktail (Biospes, China) at 4°C for 30 min using the method described by Ali et al. (2018) and then centrifuged at 10,000 × g for 10 min. The total protein concentrations were measured according to the Biuret method (WANG et al., 1996). A measure of 30 μg of protein was loaded in each lane and resolved by 10% SDS-polyacrylamide gel electrophoresis and then transferred to the PVDF membrane (Millipore, Merck, United States) using semi-dry transfer methods (Towbin et al., 1979). Five percent non-fat milk in the TBST buffer was used as a blocking solution for 1 h at room temperature, and then, the membrane was incubated overnight with primary antibodies at 4°C. The antibodies used were anti- NF-kB p65 (1:500), anti-Nrf2 (1:500), anti-SIRT1 (1:500) (Chongqing Biospes Co., Ltd., China), and anti-TNF-α (1:500) (Santa Cruz Biotechnology, Inc., sc-52746). Then, the membranes were incubated with the alkaline phosphatase-conjugated secondary antibody (Biospes, China, dilution 1:5,000) for 1 h. The BCIP/NBT substrate detection kit (Genemed Biotechnologies, United States) was used to visualize the bands. The obtained bands were analyzed using ImageJ^®^ software (National Institutes of Health, Bethesda, United States) in relation to the loading control *β*-actin bands.

### 2.8 Renal histopathology

H&E stained sections of kidneys from each group were examined by a histopathologist, in a blinded manner, under a light microscope, and the damage was assessed according to Jablonski et al. (1983)’s scoring system.

### 2.9 Statistical analysis

Data were expressed as mean ± S.E.M and evaluated using one-way ANOVA, followed by Tukey’s multiple comparison *post hoc* test. A *p*-value less than 0.05 was considered statistically significant. Prism 9 software (GraphPad Software Inc., San Diego, CA) was used for data analysis.

## 3 Results

### 3.1 Effect of DOX and incretin-based therapies (alogliptin and semaglutide) on the kidney index

**FIGURE 2:**
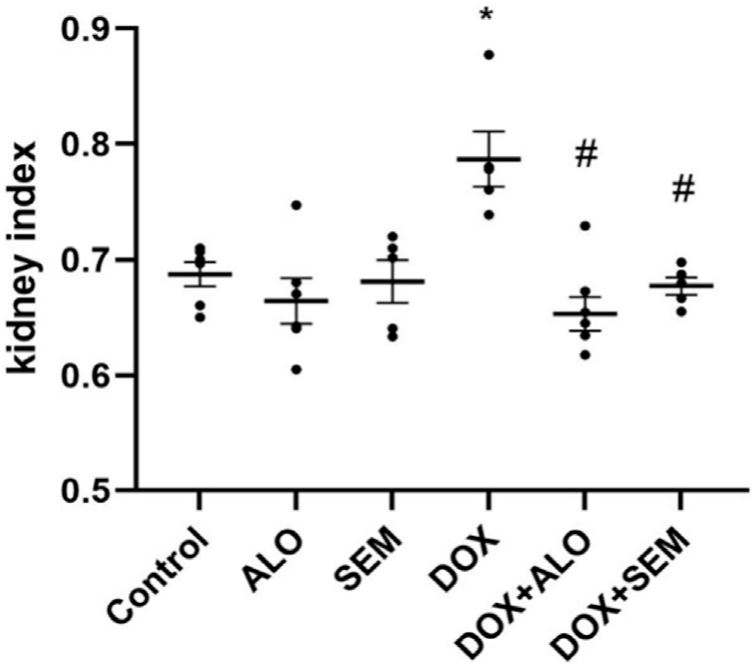
Effect of incretin-based therapies (alogliptin and semaglutide) and doxorubicin (DOX) on the kidney index. Data are represented as mean ± SEM. * is significant compared to the control group at *p* <0.05. # is significant compared to the DOX group at *p* <0.05.

Figure 2 shows that the administration of DOX, 20 mg/kg, significantly increased the kidney index compared to the control group (*p* <0.05). Co-treatment with ALO or SEM with DOX ameliorated the DOX-induced increase in the kidney index. On the other hand, there was no notable change in the kidney index among the control group, ALO alone-treated group, or SEM alone-treated group.

### 3.2 Effect of DOX and incretin-based therapies (alogliptin and semaglutide) on renal functions

**FIGURE 3:**
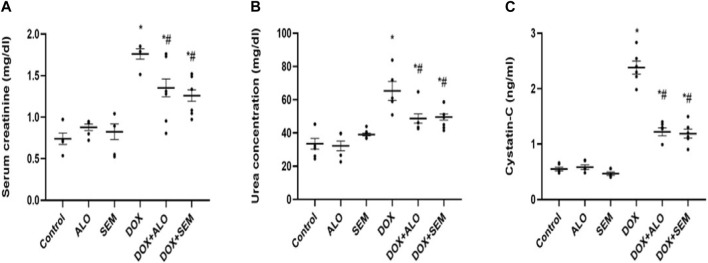
Aligned dot plots showing the effect of incretin-based therapies (alogliptin and semaglutide) on the DOX-induced renal dysfunction: **(A)** serum creatinine, **(B)** urea, and **(C)** cystatin C concentrations. Data are represented as mean ± SEM. * is significant compared to the control group at *p* <0.05, and # is significant compared to the DOX group at *p* <0.05.

Renal damage induced by DOX was evident by the significant (*p* <0.05) elevation of the serum levels of creatinine, cystatin C, and urea, compared to the control group. Co-treatment with either alogliptin or semaglutide showed significant (*p* <0.05) improvement in DOX-induced renal dysfunction, as shown in Figure 3.

### 3.3 Effect of DOX and incretin-based therapies (alogliptin and semaglutide) on renal oxidative stress markers

**FIGURE 4:**
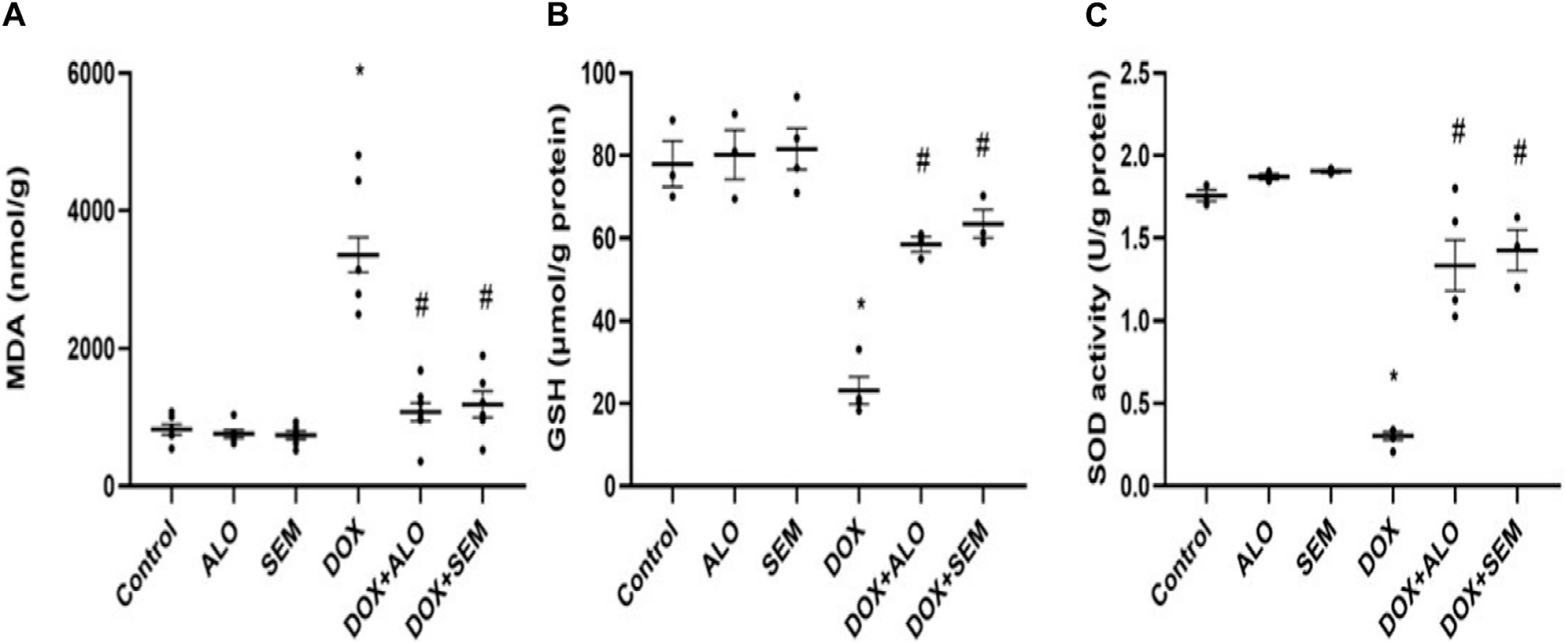
Aligned dot plots showing the effect of DOX and incretin-based therapies (alogliptin and semaglutide) on the renal levels of MDA **(A)**, GSH **(B)**, and SOD **(C)**. Data are represented as mean ± SEM. * is significant compared to the control group at *p* <0.05. # is significant compared to the DOX group at *p* <0.05.

The administration of DOX (20 mg/kg) resulted in a significant (*p* <0.05) increase in the MDA level and a significant decline in the reduced glutathione level and SOD activity in renal tissues compared to that in the control group. On the other hand, treatment with either alogliptin or semaglutide significantly (*p* <0.05) ameliorated these changes compared to the DOX alone-treated group, as illustrated in Figure 4.

### 3.4 Effect of DOX and incretin-based therapies (alogliptin and semaglutide) on the renal expression of Nrf2

**FIGURE 5:**
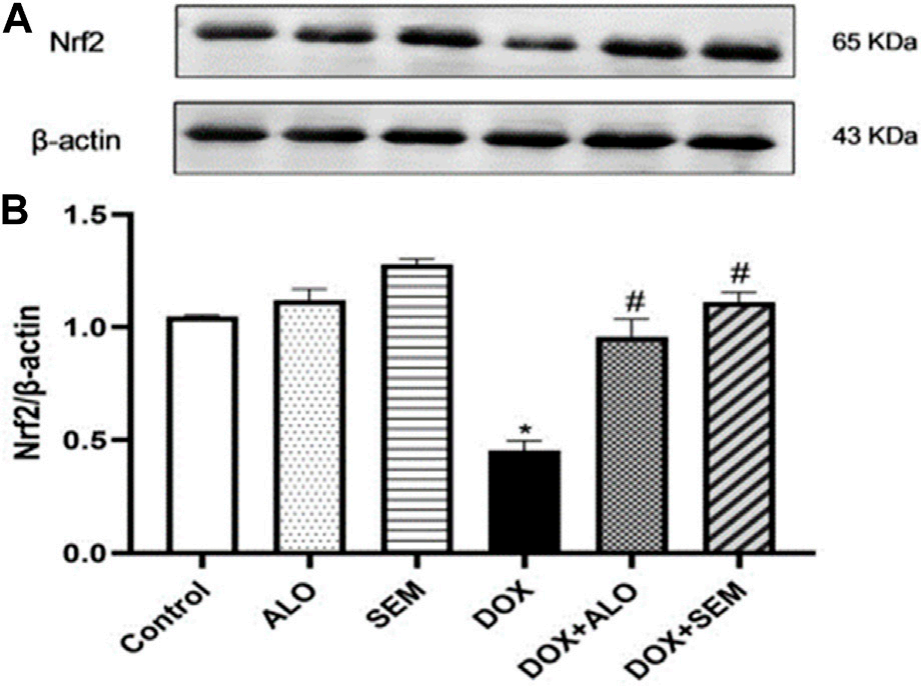
Western blot analysis of the effect of DOX and incretin-based therapies (alogliptin and semaglutide) on the renal expression of Nrf2. Nrf2 and *β*-actin bands of each group are shown **(A)**, along with the bar chart of their densitometric analysis **(B)**. Data are represented as mean ± SEM. * is significant compared to the control group at *p* <0.05. # is significant compared to the DOX group at *p* <0.05.

Figure 5 shows that the administration of DOX, 20 mg/kg, caused a significant (*p* <0.05) decline in the renal expression of Nrf2 compared to the control group, while the concomitant administration of either alogliptin or semaglutide with DOX significantly (*p* <0.05) prevented this decline.

### 3.5 Effect of DOX and incretin-based therapies on the renal expression of sirtuin 1

**FIGURE 6:**
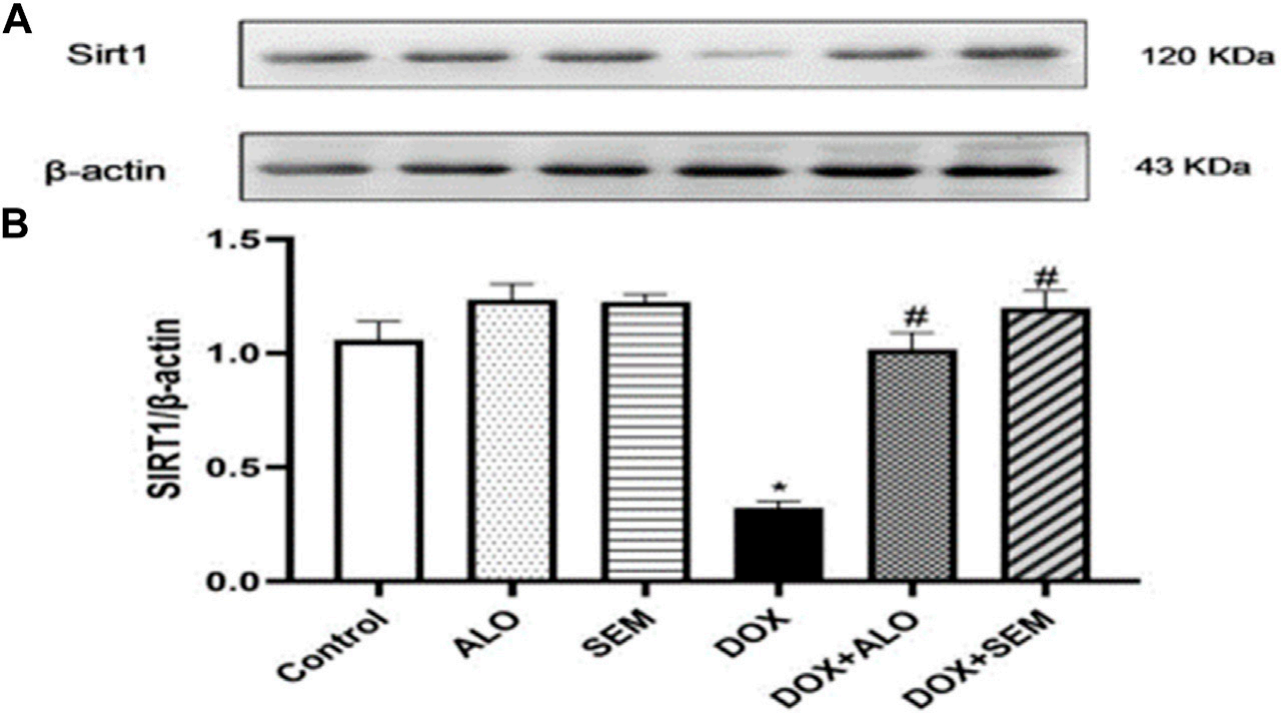
Western blot analysis of the effect of DOX and incretin-based therapies (alogliptin and semaglutide) on the renal expression of SIRT1. SIRT1 and *β*-actin bands of each group are shown **(A)**, along with their bar chart of their densitometric analysis **(B)**. Data are represented as mean ± SEM. * is significant compared to the control group at *p* <0.05. # is significant compared to the DOX group at *p* <0.05.

Figure 6 illustrates the effect of DOX administration and concomitant treatment with either alogliptin or semaglutide on the renal expression of sirtuin 1. Treatment with DOX resulted in the significant (*p* <0.05) downregulation of the sirtuin 1 expression compared to that in the control group. On the other hand, treatment with either alogliptin or semaglutide, concomitantly with DOX, significantly (*p* <0.05) prevented the DOX-induced decline in the SIRT1 renal level.

### 3.6 Effect of DOX and incretin-based therapies (alogliptin and semaglutide) on the renal expression of NF-κB

**FIGURE 7:**
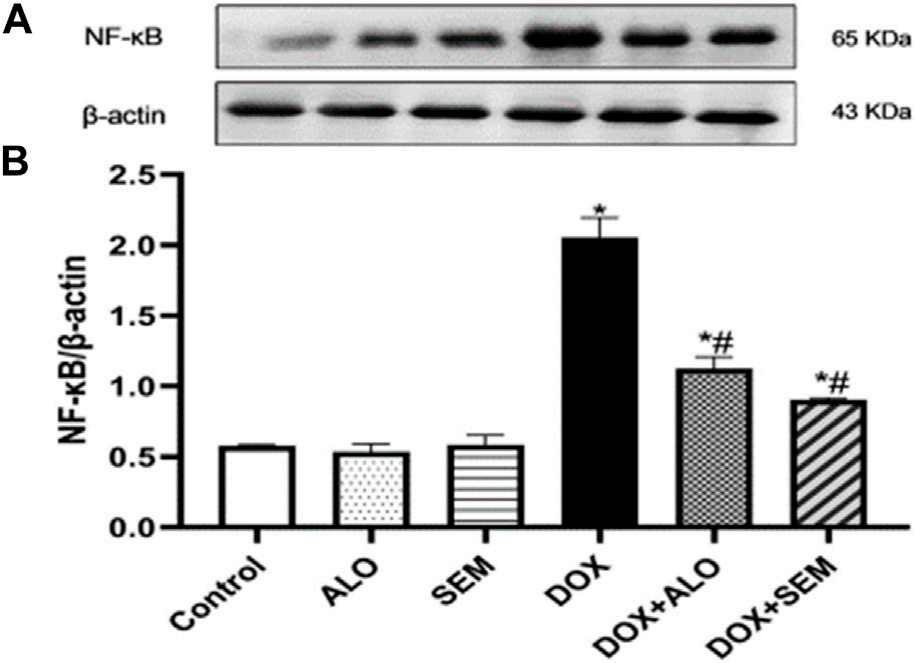
Western blot analysis of the effect of DOX and incretin-based therapies (alogliptin and semaglutide) on the renal expression of NF-κB, showing NF-κB bands and the corresponding *β*-actin bands of each group **(A)** and the bar chart of their densitometric analysis **(B)**. Data are represented as mean ± SEM. * is significant compared to the control group at *p* <0.05. # is significant compared to the DOX group at *p* <0.05.

DOX administration resulted in the significant (*p* <0.05) upregulation of the renal level of NF-κB compared to that in the control group. Co-treatment with alogliptin or semaglutide significantly (*p* <0.05) abrogated the increase in the NF-κB renal expression induced by DOX, as illustrated in Figure 7.

### 3.7 Effect of DOX and incretin-based therapies on the renal expression of the tumor necrosis factor-alpha

**FIGURE 8:**
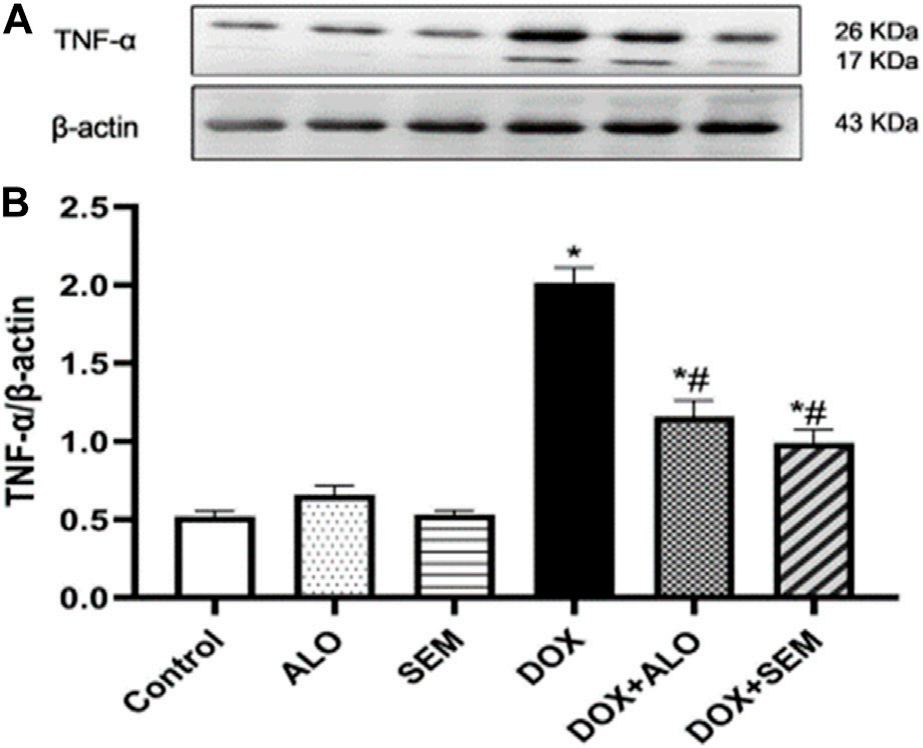
Western blot analysis of the effect of DOX and incretin-based therapies (alogliptin and semaglutide) on the renal expression of TNF-α, showing TNF-α bands and the corresponding *β*-actin bands of each group **(A)** and bar chart of their densitometric analysis **(B)**. Data are represented as mean ± SEM. * is significant compared to the control group at *p* <0.05. # is significant compared to the DOX group at *p* <0.05.

Compared to the control group, the renal expression of TNF-α was significantly (*p* <0.05) increased in the DOX-treated group. This effect was significantly (*p* <0.05) abrogated by concomitant treatment with either alogliptin or semaglutide, as shown in Figure 8.

### 3.8 Effect of incretin-based therapies (alogliptin and semaglutide) on the renal histopathological changes induced by DOX

**FIGURE 9:**
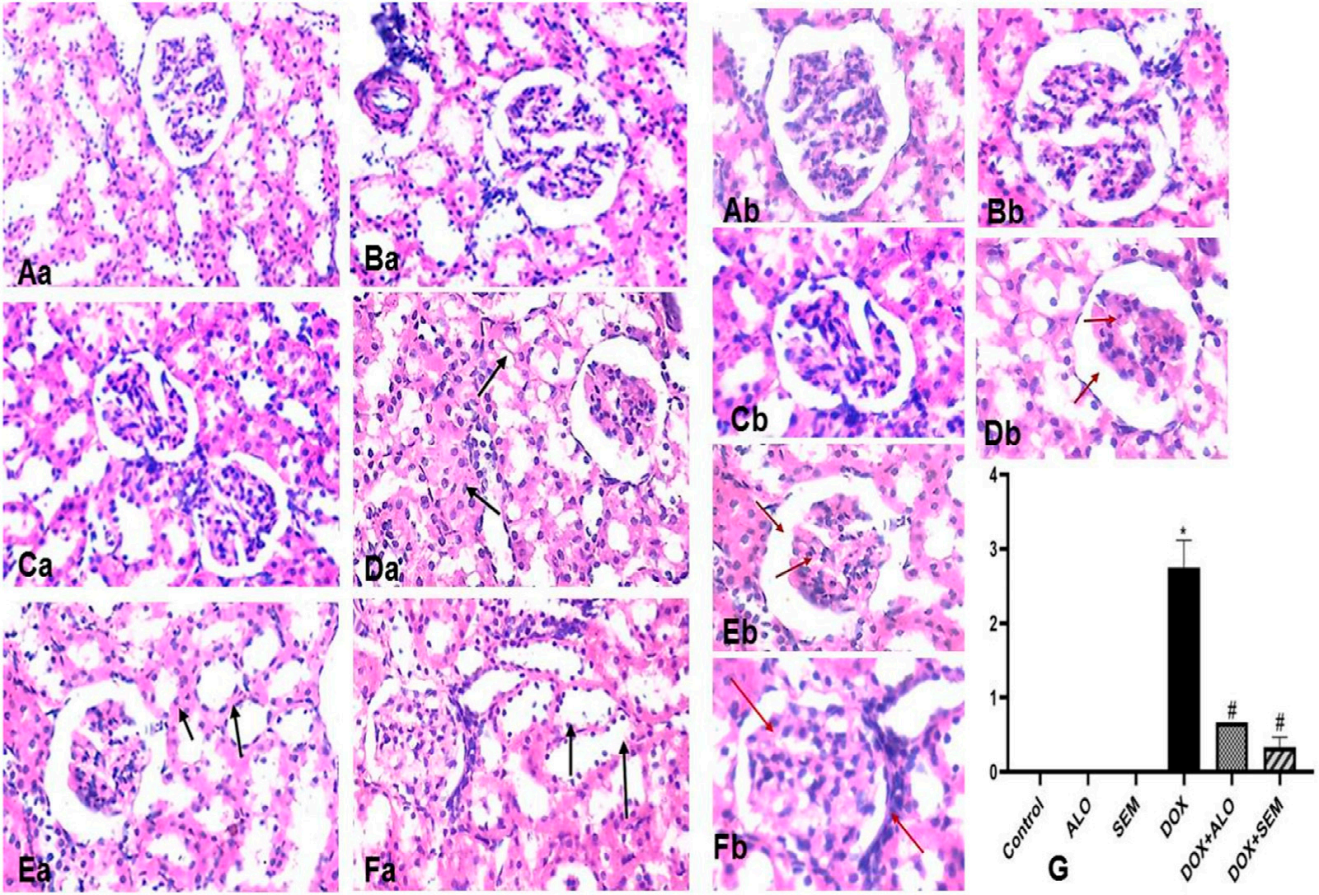
Photomicrographs of the kidney tissue sections of the control group (**(Aa)**, X200 and **(Ab)**, X400), alogliptin group (**(Ba)**, X200 and **(Bb)**, X400), and semaglutide group (**(Ca)**, X200 and **(Cb)**, X400), showing their normal histological structure; the DOX group (**(Da)**, X200 and **(Db)**, X400) shows a tubular form with necrosis with the intratubular albuminous casts (arrow) and the marked retraction of capillary tufts and the widening of Bowman’s space (red arrow). The DOX + ALO group (**(Ea)**, X200 and **(Eb)**, X400) shows the loss of the tubular epithelial brush border with the integrity of the basal membrane (arrow), along with the mild retraction of capillary tufts and the widening of Bowman’s space (red arrow). The DOX + SEM group (**(Fa)**, X200, **(Fb)**, X400) shows the intact tubular epithelial lining (arrow) and the mild thickening of Bowman’s capsule without the retraction of capillary tufts (red arrow), and the bar chart **(G)** represents the total damage score of each group. Data are represented as mean ± SEM. * is significant compared to the control group at *p* <0.05. # is significant compared to the DOX group at *p* <0.05.

The kidney tissue section of the control and ALO- and SEM-treated groups showed a normal histological structure of the renal tubular epithelial lining. No inflammation or necrosis was observed. There is no visible interstitial damage/abnormality. The blood vessels showed a uniform endothelium with no swelling or disruption of the endothelial cells. The glomerulus appeared intact with thin-walled Bowman’s capsules and no tuft retraction (Figures 9Aa,b, Ba,b, Ca,b). On the other side, the kidney sections of the DOX-treated group revealed varying degrees of tubular epithelial cell damage. Some renal tubules showed the necrosis of their epithelial lining with intratubular albuminous casts. The renal blood vessel showed the loss of the endothelial lining and perivascular edema. Glomeruli showed a marked retraction of capillary tufts and the widening of Bowman’s space. An inflammatory reaction appeared in the form of the congestion of blood capillaries and mononuclear cell infiltration of the interstitial compartment, with necrosis in up to 60% of the cells (Figures 9Da, b). Kidney sections of the DOX + ALO group showed a loss of the tubular epithelial brush border in less than 25% of tubular cells with the integrity of a basal membrane. The renal blood vessel showed endothelial swelling. Glomeruli showed the mild retraction of capillary tufts and the widening of Bowman’s space, causing inflammation and hemorrhage in less than 25% of the tissue (Figures 9Ea, b). Kidney sections of DOX + SEM showed an intact tubular epithelial lining without notable pathological alterations. The renal blood vessel showed an intact endothelial lining. Glomeruli showed the mild thickening of Bowman’s capsule without the retraction of capillary tufts and the absence of inflammation, congestion of blood capillaries, hemorrhage, and tubular necrosis (Figures 9Fa, b). Figure 9G represents a bar chart of histopathological damage scoring of each group, revealing that the administration of DOX induced significant histopathological damage, while treatment with either alogliptin or semaglutide showed improvements in DOX-induced damage.

## 4 Discussion

Doxorubicin is a widely used anthracycline antineoplastic agent; however, its renal adverse effects may limit its use (Ayla et al., 2011). The exact mechanism responsible for DOX-induced renal damage is not fully understood; however, many studies have emphasized the role of oxidative imbalance in DOX-induced nephrotoxicity (Deman et al., 2001; Lebrecht et al., 2004; Carvalho, et al., 2009; Afsar et al., 2020). DOX damages the mitochondrial DNA and decreases the activities of mitochondrial complexes I and IV, leading to mitochondrial dysfunction, accumulation of the ROS, and a rapid decline in renal functions (Okuda et al., 1986; Lebrecht, et al., 2004). In addition, studies showed that DOX administration leads to increased lipid peroxidation and a decline in renal enzymatic and non-enzymatic antioxidants (Mansour et al., 1999; Afsar, et al., 2020). These studies agreed with the observed results in this study as DOX administration resulted in increased lipid peroxidation, a lower concentration of reduced glutathione, and decreased SOD activity in renal tissues. In addition, treatment with DOX decreased the renal expression of Nrf2, a master regulator of oxidative status, which empowers the cellular defenses against oxidative stress by increasing the transcription of antioxidant enzymes, such as superoxide dismutase, glutathione peroxidase, and glutathione S-transferase (Narasimhan and Rajasekaran, 2017). Interestingly, decreased Nrf2 paralleled the decrease in the antioxidant activities in the kidney tissues. Importantly, the activity of the Nrf2/antioxidant responsive element (ARE) pathway can be enhanced by SIRT1, a mammalian sirtuin capable of deacetylating various histone and non-histone proteins with resultant antioxidant, anti-inflammatory, and antiapoptotic effects (Haigis and Sinclair, 2010; Huang et al., 2013). Thus, we hypothesized that the DOX-mediated increase in oxidative stress is related to decreased Nrf2 and SIRT1 expressions.

The observed increase in oxidative stress parameters coupled with the decline in the renal expression of Nrf2 and SIRT1 was diminished by treatment with either alogliptin or semaglutide. This agrees with the reported data by Xiang et al. (2023), who showed the activating effect of semaglutide on the SIRT1 pathway in C2C12 myotube cells. The same effect was observed using alogliptin against cyclophosphamide-induced liver injury (Salama et al., 2019). In addition, a previous study by Abdel-Latif et al. (2020) showed that low-dose lixisenatide treatment was able to protect against early diabetic nephropathy by causing a significant decrease in renal malondialdehyde and the total nitrite levels along with a marked rise in the total antioxidant capacity. On account of these findings, we supposed that the use of the incretin-based therapy concurrently with DOX could mitigate the increase in renal oxidative stress via the upregulation of renal Nrf2 and SIRT1 expressions. As a result of this oxidative imbalance, renal functions declined, and this was observed by the increase in serum creatinine and urea, as well as cystatin C levels (which are the biomarkers of kidney function that are usually monitored to assess the glomerular function). In addition, marked alterations were observed in the histological examination, including tubular necrosis, cast formation, perivascular edema, glomerular tuft retraction with the widening of Bowman’s space, congestion, and inflammatory cell infiltration, with a marked increase in the renal index of the DOX-treated group. These results agree with the previous studies (Heeba and Mahmoud, 2016; Hazem et al., 2022). Previous reports have supported the role of GLP-1RAs on diabetic kidney diseases studied until now; the mechanisms that underlie such effects are to be clarified. It has been found that GLP-1 receptors are abundantly expressed in the glomerulus and the proximal convoluted tubule in kidney tissues, which explains their role in modulating the renal physiological and pathophysiological functions (Crajoinas et al., 2011; Dieter et al., 2018). However, treatment with either alogliptin or semaglutide ameliorated DOX-induced renal damage, as manifested by the decrease in serum creatinine, urea, and cystatin C concentrations, in addition to the observed improvement in the histopathological changes in renal tubules, glomeruli, and interstitial tissues compared to the DOX-treated group.

In addition to the reported and abovementioned role for oxidative stress, some studies have suggested the role of inflammation in the pathogenesis of DOX-induced nephropathy (Zhang et al., 2017; Ibrahim et al., 2020). Their suggestion agreed with the observed upregulation of NF-κB and TNF-α in the renal tissues of the DOX-treated group in this study. It has been found that the activity of NF-κB is enhanced by various pro-inflammatory cytokines, such as TNF-α (Beg et al., 1993). Furthermore, the activation of NF-κB leads to the transcription of many genes encoding the pro-inflammatory cytokines, such as TNF-α and different interleukins (Liu et al., 2017). This crosstalk between NF-κB and TNF-α was also observed by Kagoya et al. (2014). Notably, a correlation between NF-κB and Nrf2 exists; it has also been reported that Nrf2 mitigates inflammation either by blocking the translocation and activation of NF-κB or by directly inhibiting the pro-inflammatory cytokine gene transcription (He et al., 2020). In the present study, co-treatment with either alogliptin or semaglutide ameliorated the coupled upregulation of NF-κB/TNF-α induced by DOX. These results are in accordance with other studies reporting the anti-inflammatory effects of GLP-1RA, semaglutide, in different models, such as obesity, multiple sclerosis, and diabetes (Tan and Tan, 2019; Pan et al., 2023; Sadek et al., 2023), and the DPP-4 inhibitor, alogliptin, in lipopolysaccharide-induced neuroinflammation (El-Sahar et al., 2021). It is also worth mentioning that alogliptin showed the protective effect against DOX-induced testicular dysfunction through decreasing oxidative stress, inflammation, and apoptosis (Kabel, 2018). Accordingly, it appeared that treatment with alogliptin or semaglutide succeeded in preventing the DOX-induced renal damage. The observed nephroprotective effect of alogliptin and semaglutide in this study may be mediated by the antioxidant and anti-inflammatory effects of both drugs by modulating the SIRT1/Nrf2/NF-κB/TNF-α signaling pathway, with no superiority of either drug above the other with respect to the doses used in this study. This nephroprotective effect was also observed using other DPP4is, such as sitagliptin, saxagliptin, vildagliptin and linagliptin (Jo et al., 2018; Mostafa et al., 2021), and the GLP1RA, lixisenatide (Guo et al., 2019).

In conclusion, augmenting the GLP-1 pathway either by DPP-4 inhibitors or by directly using GLP-1RAs with the resultant antioxidant and anti-inflammatory effect represents a promising strategy for preventing DOX-induced nephrotoxicity. However, further clinical studies are needed to validate the potential significance of this study and to find the proper therapeutic implementation in ameliorating the adverse effects of anti-cancer drugs.

## Data Availability

The raw data supporting the conclusion of this article will be made available by the authors, without undue reservation.
